# Variation in gene expression patterns in effusions and primary tumors from serous ovarian cancer patients

**DOI:** 10.1186/1476-4598-4-26

**Published:** 2005-07-21

**Authors:** Marci E Schaner, Ben Davidson, Martina Skrede, Reuven Reich, Vivi Ann Flørenes, Björn Risberg, Aasmund Berner, Iris Goldberg, Vered Givant-Horwitz, Claes G Tropè, Gunnar B Kristensen, Jahn M Nesland, Anne-Lise Børresen-Dale

**Affiliations:** 1 Departments of Biochemistry (M.E.S.), Stanford University School of Medicine, Stanford, CA 94305-5151, USA; 2 Department of Pathology, The Norwegian Radium Hospital, Montebello N-0310 Oslo, University of Oslo, Norway; 3 Department of Pharmacology and Experimental Therapeutics, Faculty of Medicine, Hebrew University, Jerusalem 91120, Israel; 4 Department of Gynecologic Oncology, The Norwegian Radium Hospital, University of Oslo, Montebello N-0310 Oslo, Norway; 5 Department of Genetics, The Norwegian Radium Hospital, University of Oslo, Montebello N-0310 Oslo, Norway; 6 Deceased

## Abstract

**Background:**

While numerous studies have characterized primary ovarian tumors, little information is available regarding expression patterns of metastatic sites of this cancer. To define sets of genes that distinguish primary and metastatic ovarian tumors, we used cDNA microarrays to characterize global gene expression patterns in 38 effusions (28 peritoneal, 10 pleural) and 8 corresponding primary ovarian tumors, and searched for associations between expression patterns and clinical parameters.

**Results:**

We observed multidimensional variation in expression patterns among the cancers. Coordinate variation in expression of genes from two chromosomal regions, 8q and 19q, was seen in subsets of the cancers indicating possible amplifications in these regions. A set of 112 unique genes of known function was differentially expressed between primary tumors and effusions using supervised analysis. Relatively few differences were seen between effusions isolated from the pleural and peritoneal cavities or between effusions from patients diagnosed with stage III and stage IV cancers. A set of 84 unique genes was identified that distinguished high from lower grade ovarian cancers. The results were corroborated using immunocytochemistry, mRNA *in situ *hybridization, and immunoblotting.

**Conclusion:**

The extensive variation in expression patterns observed underscores the molecular heterogeneity of ovarian cancer, but suggests a similar molecular profile for ovarian carcinoma cells in serosal cavities.

## Background

Epithelial ovarian carcinoma claims more lives than any other gynecologic malignancy, largely because it frequently escapes detection after it has metastasized [1]. Ovarian carcinoma initially metastasizes primarily to the serosal surface of the peritoneal cavity and abdominal organs. The pleural space is often involved as well, either at diagnosis or, more commonly, at later stages of clinical progression. Pleural effusion is the most common presentation of stage IV disease [2]. A number of metastasis-associated molecules have been reported to be differentially expressed between primary ovarian tumors and tumor cells in effusions [3-12], but little is known regarding the mechanism of metastases.

Molecular characterization of ovarian carcinoma using DNA microarrays has so far focused on primary tumors [13-22]. The paucity of data regarding the biological characteristics of ovarian carcinoma cells in effusions at both the phenotypic and genotypic level limits our understanding of tumor progression in this disease. Specifically, we do not know how ovarian carcinoma cells in ascites and pleural effusions differ from those in the corresponding solid primary tumors, or whether and how carcinoma cells in peritoneal and pleural effusions differ. Moreover, molecular analysis of malignant effusions might contribute to better predictions of survival and treatment response.

To identify genes whose expression may be associated with this metastatic behavior, we analyzed global gene expression patterns of ovarian cancer cells obtained from 3 distinctive anatomic sites: 28 peritoneal, 10 pleural and 8 primary tumors (see supplementary Table S1.xls). A valuable feature of this dataset is that it includes 8 paired samples of primary tumors and malignant effusions from the same patients. We were able to define a number of genes that differentiate primary tumors from effusions.

## Results

### Overview of global gene expression patterns among ovarian cancers

**Figure 1:**
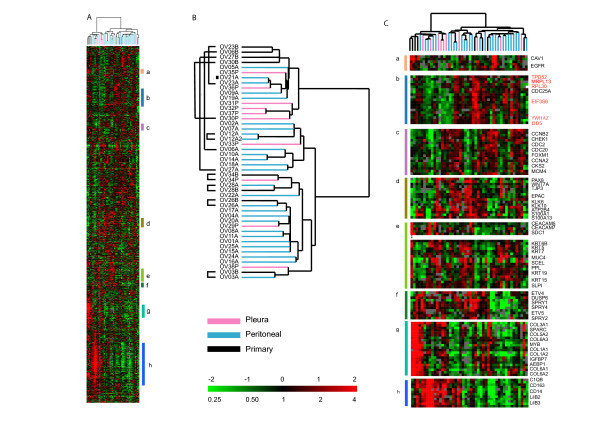
Overview of Primary Tumors and Effusions. (A) Global gene expression patterns of 46 ovarian cancers: 8 primary tumors, 10 pleural effusions and 28 peritoneal effusions, were sorted based on similarity of expression following hierarchical clustering. 2863 genes were selected from the total data set based on variance greater than 2.5 fold in at least 3 arrays. (B) The dendrogram is color-coded pink (pleural effusions), blue (peritoneal effusions) and black (primary tumors) to indicate site of origin of the cancers. Indicates the clear cell sample. (C) Magnified view of specific gene clusters selected from the entire set of 2863 genes: (a) Caveolin 1 and EGFR, (b) Chromosome 8 (Genes on Chromosome 8 are denoted in red), (c) Cell Cycle Associated Genes, (d) Kallikreins 6, 10, (e) Epithelial, (f) Sprouty cluster, (g) Stromal cluster, (h) immune response cluster. The scale is indicated in the bottom right-hand corner and spans 0.25 to 4 fold over mean (-2 to +2 in log_2 _space). Missing data are denoted in gray.

**Figure 4:**
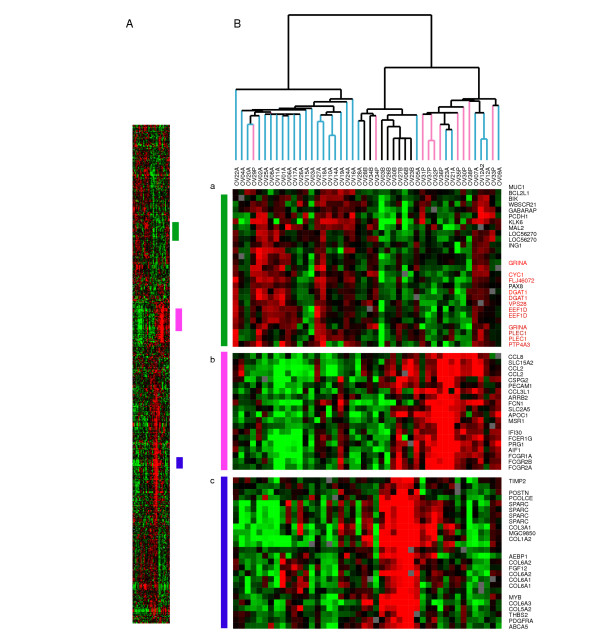
Overview of PAM results following clustering. PAM was carried out to determine differences between the 3 sites examined in this study: Primary tumors (Black), peritoneal effusions (Blue) and pleural effusions (Pink). Three main clusters differentiate the groups: (a) a set of genes that divides the cluster in to 2 groups and is more highly expressed in the majority of ascites (genes mapping to chromosome 8 are denoted in red), (b) a set of genes more highly expressed among the pleural effusions, but also expressed in a subset of the ascites, (c) Large cluster over-expressed among the primary tumors.

We profiled 46 ovarian tumor samples, 38 effusions and 8 primary ovarian carcinomas (Figure 1A–C) using cDNA arrays representing approximately 26,965 genes and selected those genes that passed a simple data quality and variation filter (see Materials and Methods). Using hierarchical clustering of the 2863 genes that passed our filtering criteria, we found considerable heterogeneity in the expression patterns among the tumor samples. The clustering analyses divided the ovarian cancer specimens into two major groups, with 4 of the 8 primary tumors clustering together but apart from their paired effusions. It is noteworthy that the other 4 primaries clustered together with the effusions from the same patient (Figure 1B). The major distinguishing feature between the two branches of the dendrogram was high expression of a number of chemokines, collagens, cell surface antigens, adhesion molecules and leukocyte antigens (Figure 1A, panels g, h). Some of the cancers were notable for the elevated expression of a cluster of genes residing on chromosome segment 8q21-24 and the coordinate variation in expression of these genes suggests that there may be an amplification of this region of chromosome 8 in some of the ovarian cancers (Figures 1C, panel b highlighted in red. See also Figure 4C, panel a). The cancers with chromosome 8q21-24 overexpression were mostly the paired primary tumors and effusions.

Genes involved in cell cycle progression and cell proliferation were variably expressed among the cancers presumably reflecting variation in proliferation rates among the tumors in a coordinated manner (Figure 1C, panel c) [23,24]. Co-expression of previously identified markers for ovarian cancer including kallikreins 6 and 10 as well as the S100 calcium-binding proteins S100A1 and S100A13 (Figure 1C, panel d) was also seen. Co-clustering of CEACAM5 and CEACAM7 with syndecan-1 (Figure 1C, panel e) was observed. These genes were part of a larger cluster with expression of Keratins 5, 6B, 7, 15 and 19, and S100A10, caveolin-2 and SLPI (Figure 1C, panel e). In another cluster (Figure 1C, panel f) the sprouty homologs: 1, 2, and 4, were co-expressed with the dual specificity phosphatase 6 (DUSP 6) and two ets variant genes 4 and 5 (ETV4 and ETV5), indicating possible involvement of the MAP kinase pathway in some tumors. Also, co-expression of caveolin 1 and EGFR was seen, in agreement with data from experimental models [25,26] (Figure 1C, panel a). The complete gene list and clustered file is found in supplementary Figure S1 CDT.cdt.

**Figure 2:**
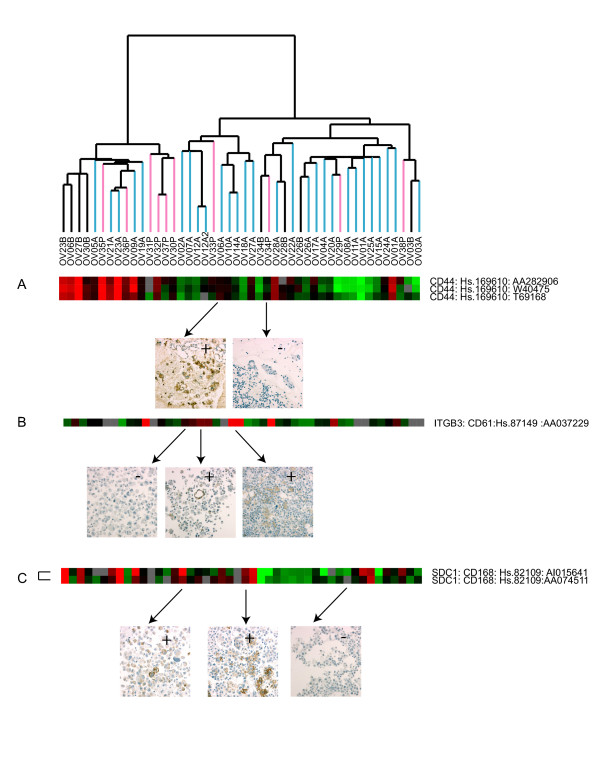
Immunocytochemistry (ICC) of selected proteins. ICC of (A) CD44, (B) CD61, and (C) Syndecan-1 (CD168) was carried out as detailed in 'Materials and Methods'. Expression Data is shown in the top panel and corresponding cases with protein staining are denoted with arrows. In panel A the following samples are shown: OV 06A and OV 34P. In panel B the following samples are shown: OV 02A, OV12A and OV14A. In panel C the following samples are shown: OV 02A, OV18A and OV20A.

In order to examine the concordance between expression at the mRNA level and protein expression, we carried out immunocytochemistry of CD44, ITGB3, and CD168 (Syndecan-1) on selected samples and show representative stains (Figure 2, panels A-C). Comparing the ICC results with the expression result gave good correlation. Of the 13 samples having both expression data and ICC for CD44, the 4 with positive ICC had a log_2 _ratio of expression from 1 to 2, and the 9 ICC negative had log_2 _ratio from 0 to -2. For the ITGB3 11 samples had both ICC and expression data, and of the 3 ICC positive the log_2 _ratios were from 1 to 2. Of the 8 ICC negatives 7 had expression ratios from 0 to -2 and one did not correspond with the expression level with a log2 ratio 1.5. For the Syndecan 1 (CD 168) of the 9 ICC positive samples 4 had log2 ratios from 1.5 to 2, 4 had log_2 _ratios of 0 and 1 had a log_2 _value of -0.5. The 3 ICC negatives had expression ratios from -1 to -2.

**Figure 3:**
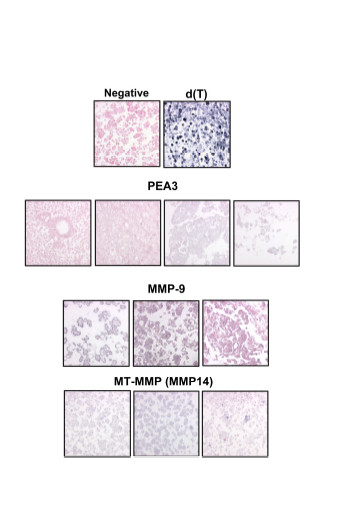
In situ hybridization of selected genes. mRNA *in situ *hybridization in ovarian carcinoma effusions. Negative control specimen (stained with nuclear fast red) and d(T) control are shown in the first row. Two negative (left, stained red) and two positive (right, gray) cases using the PEA3 probe are shown in the second row. Two positive (left, stained gray) and one negative (right, red) cases using the MMP-9 probe are shown in the third row. Three positive cases using the MMP14 probe are shown in the fourth row.

In addition, localization to the tumor cells was confirmed. In situ hybridization was carried out for the ets variant ETV4 (PEA3) (23 cases) as well as MMP-9 (19 cases) and MMP-14 (13 cases), demonstrating high mRNA expression in the tumor cells (Figure 3). These molecules were chosen since they have been shown to be involved in the metastatic process and are expressed in ovarian carcinomas and to be of prognostic value. Negative and positive controls for ICC and ISH showed consistent results through all experiments.

### Expression differences between primary tumors, peritoneal effusions and pleural effusions

To capture any differences in expression profiles between primary tumors versus all effusions, SAM analysis was performed on 2863 genes that passed the described filter criteria (see Materials and Methods). By this analysis a set of 112 unique genes of known function that were differentially expressed in these two groups was identified. (False discovery rate 2%). A partial list of the unique genes is shown in Table 1 and the complete list may be found in supplementary Table S2.xls. The relative levels of expression of several epithelial markers, including claudin 7 and keratins 7 and 19 were higher in the effusions. The expression of some genes characteristically expressed in stromal cells, including SPARC and collagens 1A1, 5A2, and 6A2 was generally higher in the primary tumors than in the effusions.

**Table 1:** Selected genes identified by SAM that are differentially expressed in primary tumors vs. all effusions. The complete list is available in Table S2.

**Genes more highly expressed in Effusions**

CLDN7 : claudin 7 : Hs.278562
KRT7 : keratin 7 : Hs.23881
CRIP1 : cysteine-rich protein 1 (intestinal) : Hs.423190
L1CAM : L1 cell adhesion molecule MASA: Hs.1757
ADM : adrenomedullin : Hs.394
CRYAB : crystallin, alpha B : Hs.391270
IL18RAP : interleukin 18 receptor accessory protein : Hs.158315
KRT19 : keratin 19 : Hs.182265
**Genes more highly expressed in Primary Tumors**
COL6A3 : collagen, type VI, alpha 3 : Hs.80988
COL1A2 : collagen, type I, alpha 2 : Hs.179573
MYB : v-myb myeloblastosis viral oncogene homolog (avian) : Hs.1334
BGN : biglycan : Hs.821
IGFBP7 : insulin-like growth factor binding protein 7 : Hs.119206
SPARC : secreted protein, acidic, cysteine-rich (osteonectin) : Hs.111779
AEBP1 : AE binding protein 1 : Hs.118397
APOD : apolipoprotein D : Hs.75736
CDH11 : cadherin 11, type 2, OB-cadherin (osteoblast) : Hs.75929

The filtered data for the effusions only were then used to examine gene expression differences among pleural and peritoneal effusions. Using SAM only 19 unique genes of known function were identified that significantly varied between pleural and peritoneal effusions (Table 2), with a false discovery rate of 13.5%. Pleural effusions had significantly higher expression of the angiogenic inducer CYR61, RAB21, glutathione S-transferase A4 (GSTA4), and several chemokines when compared to peritoneal effusions. In addition, expression of the iron transporter, SLC40A1 was generally lower in pleural effusions than in the ascites samples. Although some differences in expression were seen between pleural and peritoneal effusions, the results provide evidence in favor of a more similar genetic profile for cancer cells at these two anatomic sites than for effusions versus the primary tumor.

**Table 2:** Genes identified by SAM that are differentially expressed in Pleural vs. Peritoneal Effusions

**Positive Significant Genes: Higher in pleural vs. ascites**

NR4A1: nuclear receptor subfamily 4, group A, member 1: Hs.1119
CYR61: cysteine-rich, angiogenic inducer, 61: Hs.8867
CXCL2: chemokine (C-X-C motif) ligand 2: Hs.75765
RAB21: member RAS oncogene family: Hs.184627
CTGF: connective tissue growth factor: Hs.75511
CXCL3: chemokine (C-X-C motif) ligand 3: Hs.89690
TCEB3: transcription elongation factor B (SIII): Hs.155202
IGLL1: immunoglobulin lambda-like polypeptide 1: Hs.348935
CTGF: connective tissue growth factor: Hs.75511
IGHG3: immunoglobulin heavy constant gamma 3: Hs.413826
C1QB: complement component 1, q subcomponent beta: Hs.8986
CYR61: cysteine-rich, angiogenic inducer, 61: Hs.8867
C1QG: complement component 1, q subcomponent gamma:Hs.94953
TAGLN: transgelin: Hs.433399
CD163: Hs.74076
GSTA4: glutathione S-transferase A4: Hs.169907
**Negative Significant Genes: Lower in pleural vs. ascites**
N33: Putative prostate cancer tumor suppressor: Hs.71119 100791
PLEC1: plectin 1, intermediate filament binding protein 500 kDa: Hs.79706
SLC40A1: solute carrier family 40, member 1: Hs.5944

We then applied a supervised statistical method, PAM [27] to see whether it was possible to find a set of genes that could classify the primary tumors from peritoneal and pleural effusions. By comparing the three groups, a set of genes (615; 436 unique genes) was identified that correctly classified the primary tumors and most of the peritoneal (error rate 10%) and pleural effusions (error rate 20%). Cross-validation was not as successful, suggesting again that the difference between the 2 groups of effusions is not as clear as the distinction between all effusions and the primary tumors, which is in accordance to the results of the SAM analyses. Some differences were however detected. Clustering of the 615 genes (Figure 4 and supplementary Tables S3a, S3b.xls) illustrates that the expression of a number of chemokine ligands, including CCL2, CCL8 and CCL3L1 was more frequent in pleural effusions possibly reflecting the larger number of leukocytes in these specimens (Figure 4C, panel b). Higher expression of a number of genes on Chromosome 8q24 was observed among most of the ascites (Figure 4C, panel a highlighted in red). In addition, the expression of genes whose proteins have previously been shown to be produced by both ovarian carcinoma and stromal cells [28-30], including TIMP-2, vimentin and basic fibroblast growth factor separated the primary tumors from the effusions, suggesting that the cancer-stroma crosstalk is associated with different biological pathway activation than that observed in effusions (Figure 4C, panel c and supplementary Table S2.xls).

Hierarchical clustering of all 38 effusions was carried out as described previously. Results were very similar to those obtained when clustering both the primary tumors and effusions (Web Supplement, Figure S2.pdf) with the same 11 samples residing on the left branch as in Fig. 1 and with a strong overexpression of the immune response cluster of genes (Figure 1h). Interestingly, the majority of the pleural effusions (6/10) clustered to this branch (p = 0.02).

### Identification of Expression Profiles Based on 'Intrinsic' Genelist

**Figure 5:**
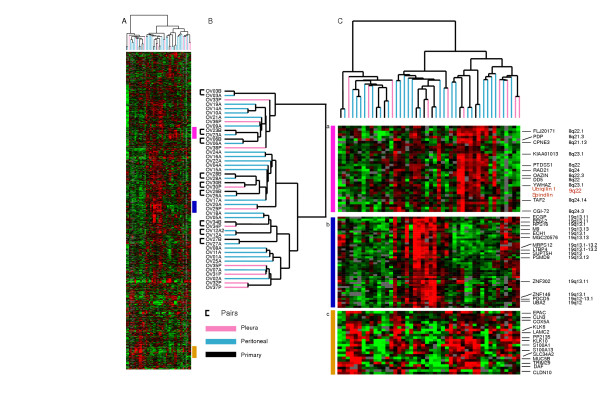
'Intrinsic' cluster: Overview of hierarchical clustering with the 'Intrinsic' genelist Overview of 2121 genes selected and magnified view of specific clusters. (a) Chromosome 8 associated cluster, (b) Chromosome 19 associated cluster, (c) Variation in expression of kallikreins and other genes among the cancers.

**Figure 6:**
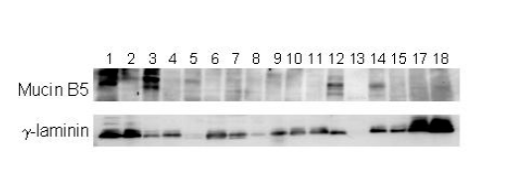
Western blotting of selected proteins. Immunoblotting of 18 effusions using antibodies directed against Mucin B5 and the laminin γ-chain (Santa Cruz). Mucin B5 (upper panel) is expressed in the specimens in lanes 1,2,3,5,12 and 14. The laminin γ-chain is expressed in all specimens except those in lanes 5 and 13. The specimens analyzed were from1 to 17: OV08A, OV09A, OV10A, OV12A, OV11A, OV02A, OV04A, OV29P, OV06A, OV01A, OV32P, OV14A, OV12A2, OV18A, OV20A, Additional specimen not on array, OV37P.

An 'intrinsic' genelist was constructed to further analyze the data (see *Materials and Methods*). The main clusters identified using this genelist highlighted probable regions of chromosomal changes on chromosomes 8 and 19 in a subset of tumors (Figure 5C, panels a, b). The majority of genes in each respective cluster mapped to either 8q21-24 or 19q13. In this analysis, all effusion-primary tumor pairs clustered together as expected. Furthermore, multi-dimensional variation was notable in groups of kallikreins 6 and 10, S100A1, S100A13, EPAC, laminin γ2 (LAMC2), MUC5B, TRIM 29 and claudin 10 (Figure 5C, panel c). The kallikreins have been shown to be potentially useful prognostic markers in ovarian cancer [31,32] and laminin γ2 and MUC5B were shown to display high expression among some of the cancers at the protein level as well, using Western analyses (Figure 6). Correlation between the protein expression and mRNA expression showed that 3 of the 4 effusions positive for MUC5B protein had positive log2 ratios. All the cases lacking protein expression had negative log2 ratios. For the laminin γ2 chain, the protein expression level did not correspond that well with the mRNA expression although all the protein-negatives had negative log2 ratios and the majority of the strongly positive effusion had positive mRNA expression.

The relationship between FIGO stage and gene expression patterns was examined using the genes identified in the intrinsic list. Using SAM, we identified only 7 named genes and one undefined clone (False Positives, 2.9) that were more highly expressed in effusions from late stage (Stage IV) ovarian cancer. Among these genes were PEN2 (presenilin enhancer 2) and PDCD5 (programmed cell death 5) both residing on 19 q12-13, (Web Supplement, Table S4.xls). Since most of the stage IV cases in this study were defined as such by the presence of pleural effusion, this further underscores the few differences between cells in effusions in Stage IIIc and Stage IV disease. We identified 84 genes that distinguished low from high grade disease (False Discovery Rate of 12.9%, Table 3, Web Supplement, Table S5.xls) including MAGE A6, a member of a family of proteins that may be useful at selectively distinguishing cancer cells from normal cells that do not express this antigen. These genes may be important in understanding the progression of ovarian cancer.

**Table 3:** representative list is shown below and the complete list is available in Table S5.


DERP6: S-phase 2 protein: Hs.417029
VARS2L: valyl-tRNA synthetase 2-like: Hs.102910
SC5DL: sterol-C5-desaturase (ERG3 delta-5-desaturase homolog, fungal)-like: Hs.287749
MYLIP: myosin regulatory light chain interacting protein: Hs.443793
HMGN3: high mobility group nucleosomal binding domain 3: Hs.77558
MAGEA6: melanoma antigen, family A, 6: Hs.441113
ATP5L2: ATP synthase, H+ transporting, mitochondrial F0 complex, subunit g, isoform 2
GPR48: G protein-coupled receptor 48: Hs.160271
MASK: multiple ankyrin repeats, single KH-domain (MASK) homolog: Hs.528646
PRO1853: hypothetical protein PRO1853: Hs.433466
YKT6: SNARE protein Ykt6: Hs.296244
ZNF-kaiso: kaiso: Hs.143604
NCK1: NCK adaptor protein 1: Hs.54589

## Discussion

We examined global gene expression patterns in primary ovarian cancers and malignant effusions and found differences that may be related to tumor progression. Our results are consistent with many reports that document the heterogeneity of gene expression patterns noted in ovarian cancer [14,16-18,20-22]. These data underscore the heterogeneity of this disease and the profound molecular differences within tumor sub-groups with comparable morphology.

Several clusters of variably expressed genes may have relevance to the biology of ovarian cancer. A large cluster of genes from chromosome 8q21-24 was more highly expressed among a subset of the cancers, suggesting an amplification of this region on chromosome 8. This was seen in both the primary and the effusions from the corresponding samples with these abnormalities. Transcripts over-expressed in this cluster include YWHAZ, that encodes the zeta isoform of 14-3-3 protein (tyrosine 3-monooxygenase/tryptophan 5-monooxygenase activation protein) family. 14-3-3 proteins are expressed in a number of cancers and are involved in the cell cycle and also in prolonging cell survival. TPD52 (hD52) that has been shown to be in the peak of the 8q21 amplicon in breast cancer cell lines was also over-expressed in a number of cancers in this cluster. A recent report suggests that TPD52 is a candidate target gene and putative oncogene at 8q21 [33]. In addition, co-clustering of a number of genes in this region was also observed in the cluster resulting from the intrinsic genelist (Figure 5C, panel a).

Involvement of the MAP kinase pathway may be indicated in a large number of the cancers in our study based on the co-expression of the 3 sprouty transcripts (1, 2, and 4) (Figure 1C panel f). Expression of all three sprouty homologs has been observed before in mouse development [34], but not in human cancers. Human sprouty 2 has been shown to inhibit the mitogen-activated protein (MAP) kinase pathway [35], and sprouty proteins have been implicated in the negative regulation of the receptor tyrosine kinase-induced MAP kinase pathway. DUSP, another gene in our sprouty cluster, belongs to a family of 9 cytoplasmic and/or nuclear enzymes, that function by dephosphorylating threonine and tyrosine residues of p38, JNK and ERK [36,37]. The transcriptional regulation of Ets transcription factors, including that of PEA3 (ETV4), on the other hand, involves signals initiated by growth factor signaling through tyrosine kinase receptors that may be mediated by MAPK [38]. We have previously shown that PEA3 and DUSP member PAC-1 predict poor outcome in ovarian carcinoma, whereas expression of all three MAPK predicts improved survival [39-41]. The coordinated expression of 3 sprouty family members, DUSP6, and ETV4 and 5 in a subset of ovarian cancers therefore raises the possibility that the activity of a specific, MAPK-related signaling pathway may have a role in ovarian cancer.

We have previously shown that clinical and molecular markers that are of established prognostic role in primary ovarian cancer have little or no significance in effusions [42]. Besides the obvious fact that cells at this site represent tumor progression, they also appear to be biologically different than tumor cells in both primary tumors and solid metastases. Using PAM to analyze the three different groups, primary tumors, and pleural and peritoneal effusions, we identified a subset of genes that is more highly expressed in the primary tumors, but fewer differences between pleural and peritoneal effusions. These genes are mainly characteristic of previously defined 'stromal' signature [43], but that are produced by both tumor and stromal cells including collagens, TIMP2, bFGF, vimentin and SPARC [28-30]. To study tumor progression we evaluated the expression levels in relation to clinical parameters using SAM, focusing on FIGO stage and grade. The most notable distinctions were based on grade rather than stage.

A large cluster of genes on chromosome segment 19q13.1 showed covarying expression among the cancers in this study, consistent with previous reports that the 19q13.1 region is amplified in some ovarian carcinomas [44,45]. The AKT2 oncogene contained in this region of chromosome 19, has been shown to be associated with the progression of ovarian cancer [44,45]. Interestingly, we found that some of the genes in the 'chromosome 19 cluster' including PDCD5 and PEN2, were more highly expressed in the Stage IV cancers than earlier-stage supporting the notion that amplification of the 19q13.1 region and concomitant elevated expression of these genes may play a role in the progression of the disease [46]. Furthermore in a larger cohort of advanced ovarian carcinomas amplification of this region was found to be associated with poor survival (Wang et. al., manuscript to be submitted).

In summary, we have examined the relationship between primary tumors in ovarian cancer and their corresponding effusions. The most notable difference was observed in expression patterns between effusions and the primary tumors. There is significant molecular variation among the cancers. There are some hints of differences related to tumor grade and effusion versus primary tumor that will need further investigation to see whether these are significant. Finally, some consistent features of expression patterns in subsets of the cancers may suggest possible molecular alterations involved in the biology of the tumors.

## Materials and methods

### Effusion specimens

Material consisted of 38 fresh non-fixed peritoneal and pleural effusions submitted from the Department of Gynecological Oncology to the Division of Cytology, Department of Pathology, The Norwegian Radium Hospital, during the period of April 1998-May 2002. Specimens were obtained pre-operatively, intra-operatively, or at disease recurrence, from 36 patients diagnosed with ovarian carcinoma (35 of the serous type, one clear cell type) and one patient diagnosed with primary peritoneal carcinoma (PPC). From one patient, two ascites specimens were obtained one month apart. Effusion specimens consisted of 28 peritoneal and 10 pleural effusions. Patient age ranged from 35 to 73 years (mean = 58 years). Twenty patients were diagnosed with FIGO stage III disease and 16 with stage IV disease. The remaining patient had a stage I tumor. Tumor grade for 36 serous carcinomas was as follows: 5 grade 1, 10 grade 2 and 21 grade 3 carcinomas. All relevant clinical data were obtained from the Department of Gynecologic Oncology, and are presented in detail in web supplementary Table S1.xls. In order to preserve physiological activity, specimens submitted to our laboratory arrived within minutes after collection and were processed immediately. Cells were suspended and frozen in RPMI+DMSO at -70°C. Smears and cellblock sections from all specimens underwent morphological evaluation by three experienced cytopathologists, and were further characterized using immunocytochemistry with broad antibody panels against epithelial and mesothelial epitopes, as previously detailed [5,6,47]. In all specimens included in this study, cancer cells comprised 50% or more of the entire cell population based on cytology smears. All patients in this study were treated with platinum based chemotherapy according to current guidelines, and the samples were collected under an IRB approved protocol (S-01127; June 22, 2001).

### Solid tumors

We obtained samples of primary tumors from 8 of the patients whose effusions were analyzed. These were snap-frozen in liquid nitrogen upon removal and stored at -70°C. Frozen sections were obtained from all biopsies in order to evaluate the percentage of tumor cells and tissue viability. The former exceeded 50% of cells in all cases.

### RNA isolation, Labeling and Hybridization

Total RNA was isolated from effusions and solid tumors using the TRIZOL Reagent (Gibco BRL, Life Technologies). mRNA isolation from total RNA was undertaken using d(T) coated Dynabeads (Dynal, Oslo, Norway). One to two μg of experimental sample mRNA was used for labeling with Cy5-dUTP. mRNA was reverse transcribed with Superscript II (Invitrogen Life Technologies) using an oligo dT primer (Operon Technologies, Alameda, CA). Each sample was comparatively hybridized to cDNA microarrays together with a common reference labeled with Cy3-dUTP (Stratagene). Fluorescent dyes were purchased from Amersham Pharmacia Biotech, Piscataway, NJ. Hybridizations were carried out using the standard protocol described previously. [23,24]. Complete experimental details may be found at: .

DNA Microarrays: All arrays were printed at the Stanford Functional Genomics Facility. DNA clones on the microarrays are based primarily the sequence verified IMAGE clones from the Research Genetics Corporation (Huntsville, AL)  and the CGAP clone set  as well as a small percentage of custom spots and control spots. Complete details regarding the clones on the arrays may be found at . These microarrays were comprised of 41,805 elements (42 K), representing an estimated 25,695 genes as judged by the number of unique Unigene symbols.

### Data Analysis and Clustering

Data Selection: Data were analyzed by using either the GenePix 3.0 or GenePix 4.0 software (Axon Instruments). Spots with aberrant measurements due to obvious array artifacts or poor technical quality were manually flagged and removed from further analysis. A filter was applied to omit measurements where fluorescent signal from the DNA spot was less than 50% above the measured background fluorescence surrounding the printed DNA spot in both the Cy3 and Cy5 Channels. Genes that did not meet these criteria for at least 80% of the measurements across the cases were excluded from further analysis. Data were retrieved as log_2_(Cy5/Cy3). The (Cy5/Cy3) ratio is defined in Stanford Microarray Database (SMD) as the normalized ratio of the background-corrected intensities [48]. Genes whose expression level differed by more than 2.5-fold from their mean expression level in the sample set in at least 3 samples, were selected for further analysis.

### Significance Analysis of Microarrays (SAM)

SAM is a statistical approach to identify genes whose expression patterns are significantly associated with specific characteristics of sample sets [49] SAM analysis was applied to the ovarian dataset to examine differences between primary, pleural and peritoneal effusions, and to examine different clinical parameters, including stage and grade. A two-way, unpaired test was carried out comparing the two groups of interest. A 10-nearest neighbor imputation engine was applied to estimate missing data [50], and 500 permutations were carried out to compute expected values and to calibrate false positive calls.

### Prediction Analysis for Microarrays (PAM)

Prediction Analysis for Microarrays (PAM)  was carried out using the Excel version of the program. This method is applied to gene expression data to provide sample classification by 'shrunken centroids'. Data were filtered as described earlier for clustering analyses. The three groups used for analysis were defined as primary tumors, peritoneal effusions (ascites) and pleural effusions [27].

### Intrinsic Genelist

An 'intrinsic' genelist, comprising genes whose expression varied significantly more between samples from different patients than between replicate samples for the same patient, was selected based on the 8 primary tumor/effusion pairs and the paired effusion cancers using methods as described previously [23]. A score was constructed which was the average within-pair variation relative to the between-pair variation. This was the ratio of the variance of the differences, relative to the variance of the averages.

### Immunocytochemical analysis (ICC)

ICC was performed using antibodies directed against Syndecan-1 (CD138; SDC1), the integrin β3 subunit (platelet glycoprotein IIIa, antigen CD61), and CD44 antigen, all part of identified gene clusters showing variable degrees of expression among the samples. Negative controls consisted of sections that underwent a similar staining procedure, with the exclusion of primary antibody application, or that were stained with mouse myeloma protein of the same isotype as the primary antibody used. Biopsies in which immunoreactivity for the studied antigens had previously been demonstrated were used as positive controls.

### Western blotting

Frozen effusion specimens were thawed and washed twice in phosphate buffered saline (PBS). Samples were subsequently lysed in 1% NP-40, 20 mM Tris HCl (pH 7.5), 137 mM NaCl, 10% glycerol, 1 mM Phenylmethylsulfonyl-fluoride, and 1 mM Sodium Orthovanadate, with 0.02 mg/ml aprotinin, leupeptin and pepstatin and 10 μg/ml phosphatase inhibitor cocktail I. All inhibitors were from Sigma-Aldrich (Saint Louis, MO). After centrifugation, the supernatant was collected and protein content was evaluated by the Bradford assay. Twenty-five μg total protein lysate/lane was resolved by SDS polyacrylamide gel electrophoresis (7.5 or 12 % gels) and transferred on to PVDF immobilon membranes (Millipore, Bedford, MA). Successful transfer was evaluated by staining of membranes withNaphtol-blue-black (Sigma-Aldrich). Filters were blocked in TBST containing 5 % dried skimmed milk and 0.05% Tween-20 for 1 hr at room temperature. Thereafter, the filters were incubated over night at 4°C with primary antibodies diluted in TBST containing 5 % skimmed milk and 0.05% Tween-20. Primary antibodies directed against the laminin γ-2 chain (Santa Cruz Biotechnology, Santa Cruz, CA) and Muc 5B (Santa Cruz) were chosen as examples since they were part of novel clusters displaying variation in expression among the cancers. Filters were washed 3 times 10 minutes each with TBST (0.05% Tween-20). They were subsequently incubated with HRP-conjugated secondary antibody diluted 1:5000 in TBST containing 5% dried skimmed milk and 0.05% Tween-20 for 45 minutes at room temperature. Immunoreactivity was detected using the ECL-plus western blotting system (Amersham-Pharmacia)

### mRNA In Situ hybridization (ISH)

Three genes were chosen for ISH as representative examples for the evaluation of mRNA expression levels in the tumor cells themselves: MMP-14, MMP-9, and PEA3 (ETV4). The following antisense oligonucleotide probes were obtained from Research Genetics (Huntsville, AL) [1-28,51,52]:

MMP-9: 5' CCGGTCCACCTCGCTGGCGCTCCGGU 3', PEA3: 5' TGA ATT ATG AGA AGC TGA GCC G 3', MMP-14: 5' TCC ATC ACT TGG TTA TTC CTC ACC CGC C 3'.

A poly d(T)20 oligonucleotide (Research Genetics) was used to verify the integrity and lack of degradation of mRNA in each sample. The DNA probes were hyperbiotinylated. Stock dilution was prepared with a resulting equal concentration for both probes. The stock dilution was diluted with probe diluent (Research Genetics) immediately before use. Specific sense oligonucleotides were used for the evaluation of non-specific activity for each probe.

Cellblock sections (4 micron-thick) of formalin-fixed, paraffin-embedded specimens were mounted on ProbeOn Plus slides (Fisher Scientific, Pittsburgh, PA). Sectioning was performed in RNase-free water. Hybridization using the probes was carried out as previously described and by using the microprobe manual staining system (Fisher Scientific)[53]. Known positive controls were used in each hybridization reaction. These consisted of 2 ovarian carcinomas for which positive hybridization was reproducible in a previous study. Controls for endogenous alkaline phosphatase for all probes included treatment of the sample in the absence of the probe and use of chromogen alone.

## Authors' contributions

ALBD, MES, BD designed the experiments and wrote the manuscript. CGT, JBK, JMN, AB, BR, BD, collected the samples and collected and analyzed the clinical data, MES, BD, RR, VAF, IG, VG, MS performed experiments.

## Supplementary Material

Additional File 1 Figure S1: CDT file for fig 1 Click here for file

Additional File 2 Figure S2: Effusion only Cluster Click here for file

Additional File 3 Table S1: Full Clinical Data Click here for file

Additional File 4 Table S2: SAM 153 full list primary tumors vs. effusions Click here for file

Additional File 5 Tables S3a, S3b: Full list of the PAM analysis comparing the primary tumors and effusions Click here for file

Additional File 6 Table S4: Analysis of stage using SAM Click here for file

Additional File 7 Table S5: Analysis of grade using SAM (full list) Click here for file
