# Supplementary figures and images for: Variation in gene expression patterns in effusions and primary tumors from serous ovarian cancer patients
Source: Mol Cancer. 2005 Jul 21;4:26. doi: 10.1186/1476-4598-4-26 (PMC1236614; doi:10.1186/1476-4598-4-26)

A

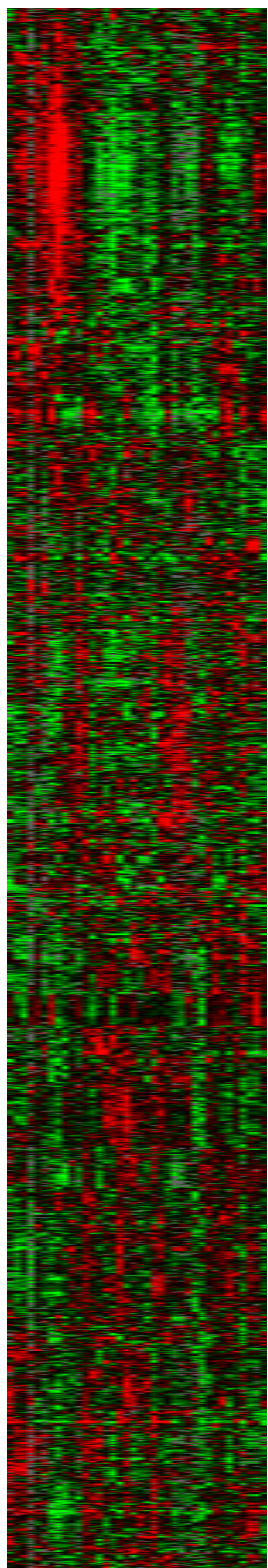

B

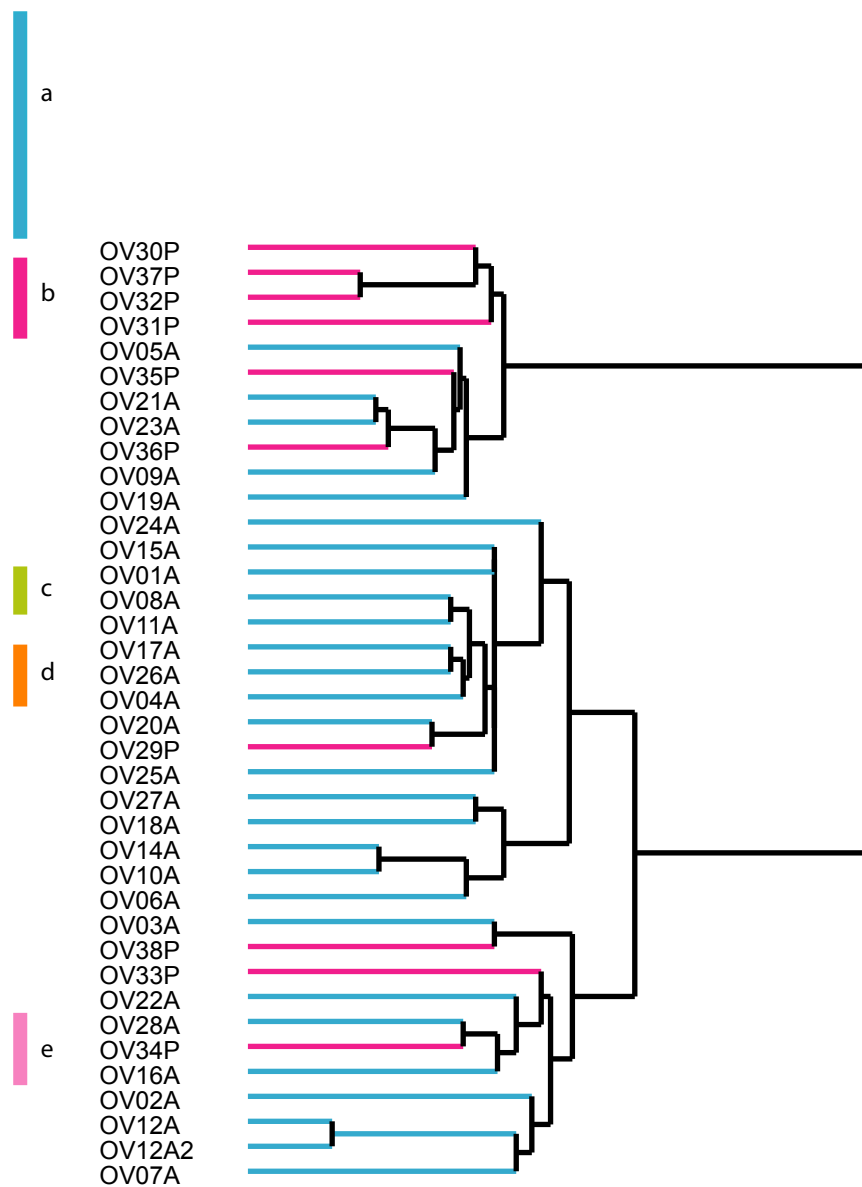

C

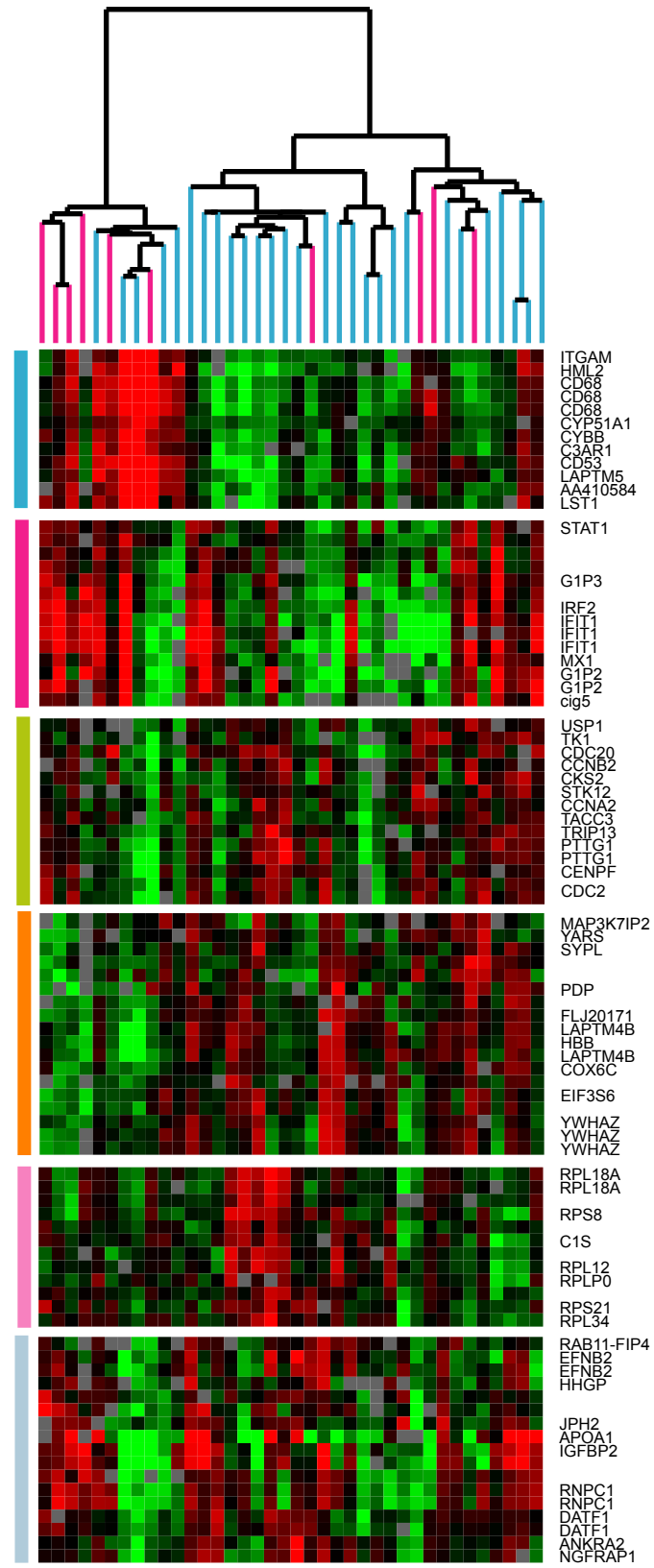

Supplement: Additional File 4 — Table S2: SAM 153 full list primary tumors vs. effusions [file 1476-4598-4-26-S4.pdf]
